# Cutaneous miliary Lymphocytoma: A Case Report with Immunohistochemical Findings

**DOI:** 10.30699/iijp.2024.2025080.3287

**Published:** 2025-03-10

**Authors:** Shatila Torabi, Sima Davoodi, Mostafa Izanlu, Pouria Etesamizade, Naser Tayebi Meibodi

**Affiliations:** 1 *Cutaneous Leishmaniasis Research Center, Department of Dermatology, * *Mashhad University of Medical Sciences, Mashhad, Iran*; 2 *Department of Pathology, Imam Reza Hospital, Mashhad University of Medical Sciences, Mashhad, Iran*; 3 *Student Research Committee, Faculty of Medicine, Mashhad University of Medical Sciences, Mashhad, Iran*

**Keywords:** Miliarial pseudolymphoma, lymphocytic infiltrates, lymphoma

## Abstract

Cutaneous pseudolymphomas (PSLs) as lymphocytic infiltrates are benign lesions that clinically and histopathologically mimic lymphomas. Miliarial type lymphocytoma cutis is an uncommon type of pseudolymphoma that is characterized by multiple semi-translucent micropapules on the sun-exposed regions. We present a 61-year-old woman who was admitted to our clinic with diffuse, sand-like, and erythematous micropapules on her face. Microscopic findings revealed nodular lymphoid aggregation, with the germinal center formation consisting of polyclonal lymphoid infiltrate confirmed by Immunohistochemistry (IHC) studies. According to this clinicopathological correlation, the miliarial type pseudolymphoma was confirmed and she was treated with topical corticosteroid ointment with a good response.

## Introduction

Skin-associated lymphoid tissue proliferation includes three categories: malignant lymphomas, atypical lymphoid proliferation (borderline lesions), and pseudolymphomas (PSLs) (1). Cutaneous PSL are benign, inflammatory, reactive, and heterogeneous T-cell or B-cell lymphoproliferative processes. The characteristic pathological findings of cutaneous lymphoid hyperplasia are germinal centers consisting of B-cell lymphocytes surrounded by T-cell lymphocytes. PSL can simulate cutaneous lymphomas histologically and clinically (2-4). This similarity makes distinguishing a malignant lymphoma from PSL challenging for dermatopathologists (5). Although PSLs are mostly idiopathic, they can sometimes occur as a result of known etiologies such as drug reactions, arthropod bites, hirudotherapy, infectious agents like Borrelia sp, injected substances, tattoos, and traumas (1, 4, 6). Clinically, PSL lesions usually appear as nodules, plaques, or noduloplaques (1). Approximately 70% of all cases present with localized nodules, mostly occurring on the face and upper limbs. However, diffuse and disseminated eruptions are rarely reported and occur predominantly in elderly individuals, affecting the midsection, limbs, and sometimes the face (7, 8). The rarity and difficulty in diagnosing this presentation of PSL prompted us to present a case with miliary PSL.

## Case Report

**Fig. 1:**
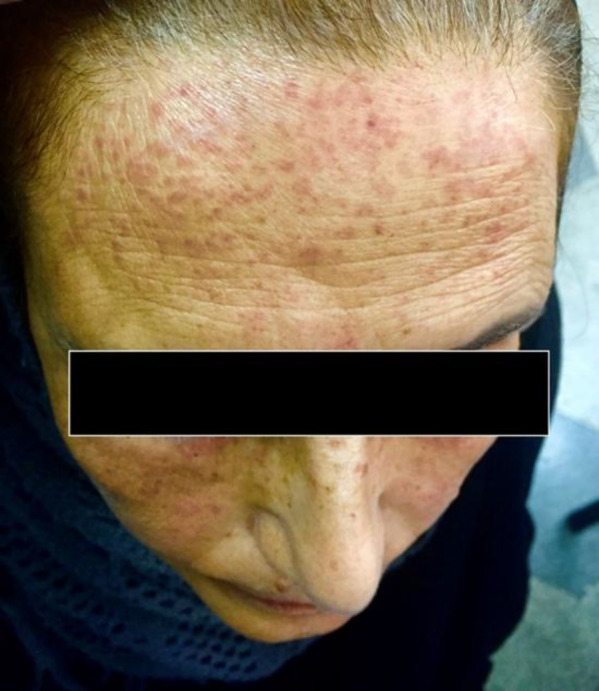
Pinpoint, miliary, and erythematous papulonodular lesions on the forehead and cheeks.

We present the case of a 61-year-old married, housewife who was admitted to the clinic of dermatology, Imam Reza Hospital, Mashhad, Iran, with diffuse, sand-like, and erythematous micro papules on her face. She reported the gradual appearance and increase of firm, pin-point and, translucent papulonodular lesions on her forehead. The lesions on her cheeks and chin showed continuous improvement. She stated that the lesions caused mild pruritus and a burning sensation when the micropapules were pressed. The lesions remained constant, and she did not indicate any association with sunlight exposure or contact with specific materials. She did not mention experiencing any hair loss, and she had no other history of skin disease or an allergic reaction to any particular material. She mentioned a history of hypertension and hyperlipidemia. She had been taking captopril 25mg once daily, aspirin 80mg once a day, and atorvastatin 40mg, one tablet each night. Furthermore, no family history of skin disease was declared. She had no signs of weight loss, fever, weakness, fatigue, organomegaly, or lymphadenopathy. During the skin examination, pinpoint, miliary, and erythematous papulonodular lesions with a diameter of 2-3 mm were observed on the forehead and cheeks. The papules did not resemble acne and had no scale, scar, pustule, comedone, or telangiectasia (Figure 1). There was no other skin manifestation on other areas of the body. Differential diagnoses could be granulomatous rosacea, acne agminata, PSL, eczema, and syringoma. Finally, a punch skin biopsy with a diameter of 0.3cm and thickness was taken from the skin of the forehead for pathological assessments.

**Fig. 2:**
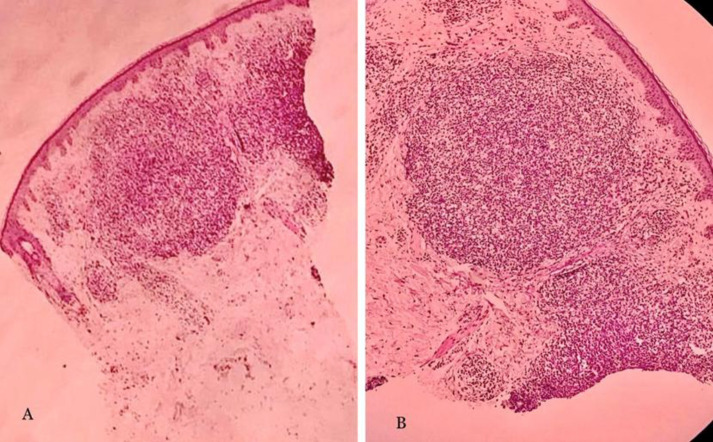
Orthokeratotic epiderm with nodular lymphoid cells aggregation with the germinal center formation and scattered tingible body macrophages was surrounded by a distinct mantle zone in the reticular dermis. (A: H and E, original magnification ×10; B: H and E, original magnification ×20)

**Fig. 3:**
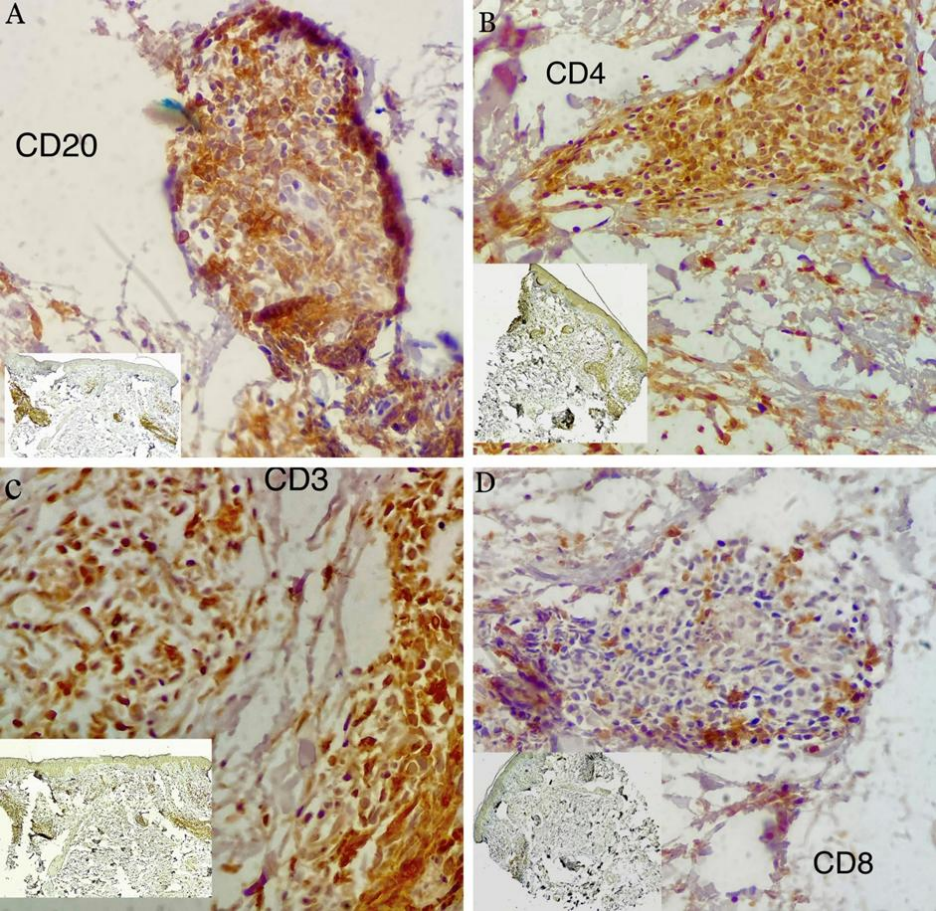
IHC staining results: (A) CD 20 is densely positive in the predominant B cell component. (B CD4 is positive in T-helper cells. (C) CD3 is positive in the concurrent T‑cell component, and (D) CD8 is positive in T-cytotoxic cells (A-C and E: original magnification ×10, D: original magnification ×4).

In the microscopic study, we observed a bulged and orthokeratotic epidermis. There were no signs of lichenoid degeneration in the basal layer. In the reticular dermis, we observed aggregation of nodular lymphoid cells with germinal center formation, as well as scattered tingible body macrophages surrounded by a distinct mantle zone. Moreover, mild chronic edema around the vessels was visible (Figure 2). Immunohistochemistry (IHC) studies were performed using CD20 (pan B cell marker), CD3 (pan T cell marker), CD4, and CD8 staining to differentiate between lymphoma and PSL. The IHC results confirmed a polyclonal lymphoid infiltrate, with CD20-positive B lymphocytes predominantly located in germinal centers surrounded by CD3-positive T lymphocytes, mostly in the mantle zones of lymphoid follicles containing helper/cytotoxic T cells (Figure 3). Additional molecular studies using polymerase chain reaction (PCR) for T-cell receptor (TCR) gene rearrangements do not show a monoclonal pattern of amplification for either TCRB or TCRG. Blood examinations, including liver function tests, complete blood count, C‑reactive protein, sedimentation rate, urea, creatinine, thyroid stimulating hormone, sodium, potassium, calcium, phosphor, and antinuclear antibody, were within normal levels. Borrelia Burgdorferi's serology was negative. Based on the pathological findings and clinic-pathological correlation, the diagnosis of miliarial type PSL was confirmed. The patient was initially treated with topical mometasone furoate ointment once daily for the first mount, followed by twice a week the subsequent months. In addition, pimecrolimus 1% was applied daily for three months, resulting in a complete response.

## Discussion

Cutaneous PSL is divided into four main groups based on histopathologic features and clinical data: (1) nodular PSL; (2) pseudo-mycosis fungoides and simulator of other T-cell lymphomas; (3) other PSL; and (4) intravascular PSL. Nodular PSL is the most common form of PSL, and in most cases, it presents as a solitary nodule. However, rarely, it can appear as disseminated papules on the face and extremities (4, 8, 9). The miliary type of lymphocytoma cutis is a rare subtype of PSL. Clinical manifestations of miliary PSL differ from other variants of lymphocytoma cutis, making it a challenging diagnostic task, particularly from a clinical perspective. The clinical features in our patient were remarkably similar, characterized by multiple semi-translucent asymptomatic 1- to 2-mm papules or a slightly pruritic rash predominantly located on the face or neck, exhibiting a symmetrical pattern. Inflammatory infiltrative patterns in cutaneous PSL can mimic cutaneous lymphoma both clinically and histologically, making it challenging to differentiate between them. The military form of primary cutaneous follicle center lymphoma (PCFL) is the primary differential diagnosis for disseminated PSL (7, 9). However, the presence of germinal centers and characteristics of IHC findings observed in disseminated PSL would not be reported in lymphoma or PCFL (7). Drugs that are commonly involved include anticonvulsants, antihypertensive, antiarrhythmic, antipsychotics, antibiotics such as levofloxacin, imatinib, cyclosporine, antirheumatics, and NSAIDs have been found to possess a higher potential for causing PSL in susceptible individuals (10, 11). In the case of our patient, the use of captopril could potentially predispose her to the PSL. 

Although the treatment of PSL can vary greatly, it is of utmost importance. The essential part of treatment is to avoid re-exposure to the causative agent. Although systemic and local treatments are available (4), it is important to note that there are paradoxical findings suggesting that PSL can progress to an overt lymphoma. Therefore, it is crucial to distinguish rare types of PSL expressions from other conditions (3). In conclusion, it is important to consider the possibility of miliary-type PSL when observing translucent, symmetrical, and asymptomatic papules on the patient's face, midsection, and extremities (7, 8). 
